# Acta Academiæ Scientiarum Imperialis Petropolitanæ, pro anno 1777

**Published:** 1781-07

**Authors:** 


IV. *Acta Academiæ Scientiarum Imperialis Petropolitanæ, pro anno* 1777. Pars prior. 4to. Petropoli, 1778, 484 pages, with 13 copper-plates.

THE nature of this work, and the views of the Academy in giving a new title to their publications, will beſt appear from the following advertiſement which is prefixed to the volume before us:

"The tranſactions of the Imperial Academy "of Sciences at St. Peterſburgh, of which "the firſt volume is now preſented to the "public,

" public, are in fact a continuation of their me-

" moirs published hitherto under the title of

" *Commentarii Academiæ Scientiarum Petropoli-*

" *tanæ*.

" From their institution in 1726 to their re-
" newal in 1747, the Academy published four-
" teen volumes. They then began a new col-
" lection, under the title of *Novi Commentarii*:
" and from 1747, to the celebration of their
" jubilee in 1776, twenty more volumes made
" their appearance, of which the xivth for 1769,
" is divided into two parts.

" The Academy perceiving that their com-

" mentaries increased in number and bulk, so

" that the purchase of them became more and

" more burthensome, resolved, in 1777, to

" change the title a third time, in order that this

" new series of their Transactions might in some

" measure form a separate work, so that the reader

" might not be obliged to load himself with the

" preceding commentaries. The Academy have

" likewise thought proper to make considerable

" alterations in the bulk, and even in the form

" of the volumes, in order to lessen the price

" and render them interesting to a greater num-

" ber of persons.

"In consequence of this resolution two vo-

"lumes of their transactions will henceforward

"appear every year, neither of which will ex-

"ceed 400 pages. In this manner the year will

"be divided into two equal portions, and each

"volume will contain the transactions of six

"months. The papers will be written in French

"or Latin, according as the authors shall think

"them more or less suited to persons not versed

"in the learned languages; and to each volume

"will be prefixed an historical part written in

"French, this language being at present the

"most generally known.

"In this historical part an exact account will

"be given of the most remarkable occurrences

"relative to the Academy, such as the discourses

"delivered at the public meetings, the questions

"proposed, and the prizes distributed by the

"Academy, the machines, inventions, and works

"presented to them; and, lastly, an abstract of

"such papers as may seem to be interesting or

"useful to the public. In order to fill up the

"void which, in consequence of this new ar-

"rangement, will be made between the last vo-

"lume of the *Novi Commentarii*, which is for

"1775, and this first volume of *Acta*, which

"is for 1777, the Academy intend to print

"an

" an index to all the volumes of their Commen-
" taries both old and new, and to give at the
" ſame time their hiſtory from their foundation
" to the celebration of their jubilee."

Agreeably to this arrangement the preſent volume conſiſts of two parts, ſeparately paged. The firſt of theſe, entitled *Hiſtoire de l'Academie*, and written in French, employs 100 pages; the remainder of the volume is allotted to the *Acta*.

In the hiſtorical part of the work, under the head of *Experimental Philoſophy*, we meet with an account of a new electrical apparatus invented by Mr. Volta, and by him named the *Perpetual Electrophorus*. It conſiſts of two plates of metal, the lowermoſt of which is covered with a layer of pitch; the other, which is ſomewhat ſmaller, is ſuſpended by ſilken cords, ſo diſpoſed by means of pullies that the plate may be brought into contact with and rubbed upon the reſinous maſs.

The electrophorus deſcribed in the volume before us was executed at St. Peterſburgh by order of the Empreſs, after a model ſent from Vienna, and is the largeſt hitherto conſtructed. It is nine feet in length, and four and a half in width. The reſinous maſs ſpread upon its under-plate contains 180 pounds of pitch, and eighty pounds pounds of Spanifh wax. In the *Acta*, Mr. Krafft gives a minute defcription of this apparatus, and a theory of its effects. This paper, which is intitled *Tentamen Theoriæ Electrophori*, is illuftrated by an engraving.

Under the head of *Natural Hiftory* we have an account of the cranium of a rhinoceros with two horns, fent to Profeffor Camper from the Cape of Good Hope, and by him prefented to the Academy. From the ftructure of this fkull Profeffor Camper is of opinion that the African rhinoceros is of a different fpecies from that of Afia.

We have next an account of fome obfervations on mufhrooms, communicated to the Academy by Father Cibot, one of their members, who is a miffionary at Pekin. This writer treats very fully of the different opinions that are to be found in the Chinefe books, concerning the nature and production of moffes and mufhrooms. The moft curious part of Father Cibot's paper is his account of the method practifed by the Chinefe for procuring a plentiful fupply of mufhrooms. It confifts, we are told, in burying rotten pieces of the wood of particular trees in a good foil, and in a fhady fituation, turned towards the fouth. The wood is fuffered to remain main in this ſtate all the winter, and is frequently watered, eſpecially when the ſummer heats have begun to take place. Nitre is diſſolved in the water employed for this purpoſe. This method, according to Father Cibot, never fails to procure muſhrooms the very firſt year.

The Chineſe are of opinion, that each tree, and even different parts of the ſame tree, produce a certain ſpecies of muſhroom which is peculiar to them. The trees they are ſaid chiefly to employ for this purpoſe, are the elm, the mulberry, the willow, the poplar, the pine, and the cheſnut.

The learned miſſionary obſerves likewiſe, that the Chineſe, who make the agarics of different trees a part of their food, have alſo a method of procuring an abundant ſupply of this kind of fungus. It conſiſts in burying the lower half of the trunk of the ſpecies of tree they have choſen (the eldeſt are ſaid to be the beſt for this purpoſe) in a ſhady place, expoſed to the ſouth. This trunk and the earth around it are to be frequently watered, eſpecially during the great heats. That part of the trunk which is above the mould, eſpecially when not deprived of its bark, is covered with agarics, which are to be ſoon gathered, otherwiſe they grow hard and woody.

woody. In a few days there is a fresh growth, and in this manner the trunk continues to produce a succession of crops for several months.

Father Cibot concludes his paper with an account of a simple method practised by the Chinese for ascertaining the salubrity of mushrooms. It consists in boiling with them a few pieces of *juncus (jonc)* the pith of which becomes of a blackish colour, if the mushrooms are of a noxious species.

The *Acta*, or second part of the volume, are divided into the three heads of *Mathematica*, *Physica*, and *Astronomica*; the papers contained in the second of these divisions, are as follows:

I. *Remarks on the natron of Russia, by* J. G. Georgi.—Hitherto native mineral alkali, which is, doubtless, the *natron* of the ancients, has been considered as a pretty rare production in the mineral kingdom. It has been occasionally found, however, in different parts of Europe, and particularly in Hungary; but no country produces it in such abundance, or so generally, as Siberia, and all the deserts of Asia.

Mr. Georgi, who has collected all the remarks made on this subject of late years by Dr. Pallas and others, is convinced by these, and by his own experiments, that this alkali is produced by the spontaneous destruction of the sea-salt and

Glauber's

Glauber's ſalt, with which the lakes and ſalt marſhes in the ſouthern provinces of Ruſſia abound. He points out the great advantages which might be derived from this natural alkali, by ſubſtituting it for the vegetable alkali in a variety of proceſſes, both in phyſic and the arts.

Towards the end of his paper he ſpeaks of a method practiſed at Nertſchinſki for procuring chryſtals from this ſalt, which anſwer all the purpoſes of borax in metallurgical operations. This proceſs our author ſuppoſes to have come originally from China, Thibet, or ſome other part of the Eaſt.

II. *A deſcription of the ledum buxifolium, by* Peter Jonas Bergius.—Botaniſts have hitherto been acquainted with only a ſingle ſpecies of ledum, that is very common in marſhy ſituations in the northern parts of Europe and Aſia. Profeſſor Bergius here deſcribes a new ſpecies found in barren ſandy ſoils in New Jerſey. This plant, of which an engraving is given in the work, differs from the common ledum in its leaves, which are oval, and ſimilar to thoſe of the *buxus*, on which account the author has given it the name of *ledum buxifolium.*

III. *A deſcription of the Digitales Hybridæ, by* I. T. Koelreuter.—The author of this paper has for

ſome time been employed in a ſeries of laborious and intereſting experiments on the production of *hybridæ* or mules in the vegetable kingdom. The laſt volume of the *Novi Commentarii*, publiſhed by the Academy, contains the reſult of his trials with different ſpecies of Lychnis, Cucubalus, Silene, and other plants of the ſame family. From theſe experiments it appears that he was able to produce *hybridæ* from the *lychnis dioica* and *cucubalus viſcoſus*, two plants of different genera.

In the preſent paper Mr. Koelreuter deſcribes the reſult of his experiments with different ſpecies of *digitalis*. The *digitalis lutea* and *digitalis purpurea* fecundated each other reciprocally, and produced real *hybridæ*, or mules, partaking of each of thoſe ſpecies. A ſimilar production took place between the *digitalis lutea*, and the *digitalis Thapſi*. Our author likewiſe procured mules by fecundating the female organs of the *digitalis ferruginea* with the male duſt of the *digitalis ambigua*; but his attempts to procure ſimilar productions by a mixture of the *digitalis lutea* and *digitalis ambigua* were unſucceſsful, a proof this that theſe two ſpecies, which by ſome botaniſts have been conſidered as ſimple varieties, are really different.

IV. A

IV. *A deſcription of the orifice of the great coronary vein*, *by* C. F. Wolff.—The great coronary vein, and the orifice by which it communicates with the right ſinus of the heart, were known to Galen, but Euſtachius ſeems to have been the firſt who noticed the valve with which this orifice is furniſhed. Since his time anatomical writers have conſtantly ſpoken of this valve as being of a ſemilunar ſhape, but the ingenious writer of the paper before us, who treats this ſubject very accurately and minutely, contends that its figure is oblong and narrow, and he conſiders it as a valve *ſui generis*, different from any other to be met with in the human body. His deſcriptions are illuſtrated by an engraving.

V. *A deſcription of the different ſpecies of phocæ*, *by* J. Lepechin.—Linnæus ſpeaks only of one ſpecies of the *phoca* or ſeal, but others, as Pontopidan and Cranzius, deſcribe four and even five kinds of this animal. Our author, who, in his voyage up the White Sea, had good opportunities for informing himſelf on this ſubject, reduces the number to two, and theſe are the *phoca oceanica*, and *phoca leporina*, of each of which he gives an accurate deſcription illuſtrated by elegant engravings.

The volume clofes with an epitome of a meteorological diary kept at St. Peterfburgh in 1776, by John Albert Euler.

